# Discovery of Novel Allosteric SHP2 Inhibitor Using Pharmacophore-Based Virtual Screening, Molecular Docking, Molecular Dynamics Simulation, and Principal Component Analysis

**DOI:** 10.3390/ph17070935

**Published:** 2024-07-12

**Authors:** Pooja Singh, Vikas Kumar, Keun Woo Lee, Jong Chan Hong

**Affiliations:** 1 Division of Applied Life Science (BK21 Four), Plant Molecular Biology and Biotechnology Research Center (PMBBRC), Gyeongsang National University (GNU), 501 Jinju-daero, Jinju 52828, Republic of Korea; poojaniper1410@gmail.com; 2 Computational Biophysics Lab, Basque Center for Materials, Applications, and Nanostructures (BCMaterials), Buil. Martina Casiano, Pl. 3 Parque Científico UPV/EHU Barrio Sarriena, 48940 Leioa, Spain; vikaspathania777@gmail.com; 3 Korea Quantum Computing (KQC), 55 Centumjungang-ro, Haeundae, Busan 48058, Republic of Korea; 4 Angel i-Drug Design (AiDD), 33-3 Jinyangho-ro 44, Jinju 52650, Republic of Korea

**Keywords:** SHP2, receptor-based pharmacophore modeling, molecular docking, molecular dynamic simulations, cancer, MM-PBSA, PCA

## Abstract

SHP2 belongs to a cytoplasmic non-receptor protein tyrosine phosphatase class. It plays a critical role in the development of various cancers, such as gastric cancer, leukemia, and breast cancer. Thus, SHP2 has gained the interest of researchers as a potential target for inhibiting tumor cell proliferation in SHP2-dependent cancers. This study employed pharmacophore-based virtual screening, molecular docking, molecular dynamic (MD) simulations, MM/PBSA, and principal component analysis (PCA), followed by ADME prediction. We selected three potential hits from a collective database of more than one million chemical compounds. The stability of these selected hit–protein complexes was analyzed using 500 ns MD simulations and binding free energy calculations. The identified hits Lig_1, Lig_6, and Lig_14 demonstrated binding free energies of −161.49 kJ/mol, −151.28 kJ/mol, and −107.13 kJ/mol, respectively, compared to the reference molecule (SHP099) with a ΔG of −71.48 kJ/mol. Our results showed that the identified compounds could be used as promising candidates for selective SHP2 allosteric inhibition in cancer.

## 1. Introduction

**Figure 1:**
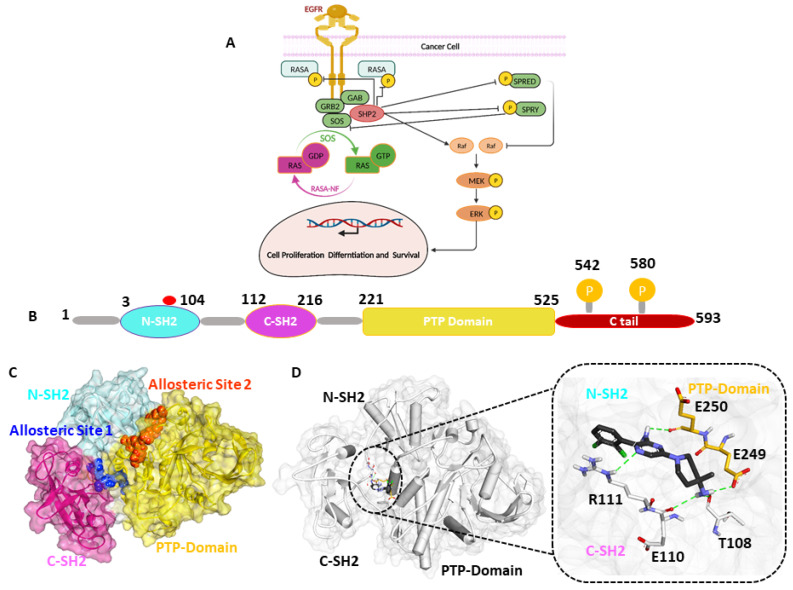
(**A**) The Ras/Raf pathway induces cell survival and proliferation due to the downstream signaling of SHP2. (**B**) A schematic representation of the SHP2 protein’s domains. The widely occurring mutation is highlighted with a red circle; among them, the E76K /D mutation was significantly observed in colorectal and breast cancer. (**C**) In the surface model of SHP2, the promising allosteric sites responsible for SHP2 inhibition are highlighted in blue and orange. (**D**) The key residues responsible for interacting with the known allosteric inhibitor SHP099 are shown in the stick model.

The Src homology protein tyrosine phosphatase 2 (SHP2) is a 595 amino acid residue containing non-receptor protein tyrosine phosphatase (PTPase) encoded by the PTPN11 gene [1,2]. SHP2 is critical in multiple signaling cascade pathways, including Ras/MAPK, JAK/STAT, and PI3K/AKT. SHP2 is also a well-known downstream effector of the programmed cell death protein 1 (PD-1) immune checkpoint receptor and regulator of immune cell function in the tumor microenvironment. The SHP2 protein consists of two SH2 domains (N-SH2 and C-SH2) present in the N-terminal and a PTP domain with two tyrosine residues (Y542 and Y580) as phosphorylation sites in the C-terminal [3,4]. N-SH2 is a key regulator of conformational switch in the activation of SHP2. The N-SH2 domain contains 2–104 amino acids. On the contrary, the C-SH2 domain contains 112–215 residues responsible for the binding energy of SHP2. The PTP catalytic domain comprises 220–525 residues, of which C459 is a highly active conserved amino acid with essential catalytic functions [5,6]. The phosphorylation of C-terminal tail residues (Y542 and Y580) occurs upon extracellular stimulation upon the binding of phosphorylating ligands like growth factors or cytokine (Figure 1). SHP2 maintains an auto-inhibited state by the intermolecular interaction of the N-SH2 and PTP domain at the basal level with deficient catalytic activity [5]. The dysregulation of the delicate balance between the phosphorylation and dephosphorylation of signaling molecules mediated by protein tyrosine kinases and protein tyrosine phosphatases (PTPs), respectively, is a distinctive feature of many cancers [7]. Mutations in the PTPN11 gene that encodes SHP2 have been associated with various human diseases, primarily through their effects on signaling pathways. Noonan Syndrome (NS) and Noonan Syndrome with Multiple Lentigines (NSML) are genetic disorders characterized by distinctive facial features, short stature, heart defects, and other developmental abnormalities [5,8]. These mutations can lead to an aberrant activation of signaling pathways, such as the RAS-MAPK pathway, contributing to uncontrolled cell growth and malignancy. Additionally, the downstream signaling of SHP2 induces cell survival and proliferation due to the Ras/Raf pathway [9,10,11]. In addition to leukemia, SHP2 mutations have been studied in various solid tumors, including lung, breast, and colorectal cancer [10,11,12,13]. The most common mutation in various tumors is SHP2E76K/D, which is crucial for increasing SHP2’s basal phosphatase activity by 20-fold [8,14]. Previous studies suggested that the role of SHP2 mutation in various diseases and cancers depends on the position and function of mutation, as E76K and E76D are the most common and active mutations observed in tumor cases [15].

The PTP domain of SHP2 is positively charged, and it has been proven previously that targeting the active site of SHP2 for developing selective inhibitors is more difficult than targeting the allosteric site [16]. Various allosteric inhibitors are still in clinical trials; SHP099 is a selective allosteric inhibitor that inhibits SHP2 at its auto-inhibited conformation and blocks its phosphatase activity [14,17,18].

In the present study, our efforts to discover novel allosteric site1 SHP2 inhibitors began with the building receptor-based pharmacophore model using SHP2E76Dprotein (PDB:6CMR) in complex with selective allosteric inhibitor SHP099 [19]. The pharmacophore model was further validated using the Güner–Henry (GH) approach and utilized for the virtual screening of four databases to discover the drug-like compounds. Then, the virtually screened compounds were docked at the allosteric site1 of SHP2, followed by 500ns molecular dynamic simulations and MM-PBSA calculation. Our findings suggest that the obtained hit compounds named Lig_1, Lig_6, and Lig_14 have the potential to inhibit SHP2 and can be useful for developing novel allosteric SHP2 inhibitors in cancer (Figure 1).

## 2. Results

**Figure 2:**
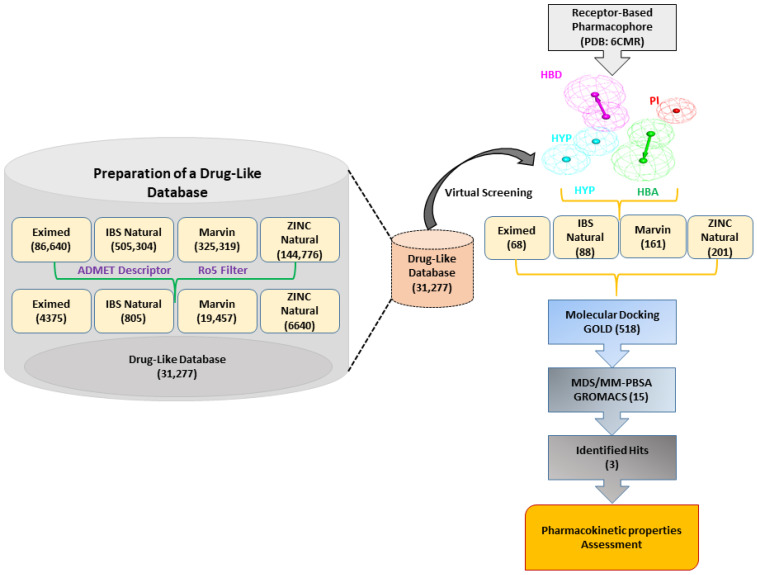
The general workflow used in the present research is for the identification of allosteric SHP2 inhibitors. Receptor–ligand-based pharmacophore generation from the PDB: 6CMR. The drug-like database generation step is shown on the left side of the image.

In this research, we implemented a structure-based pharmacophore modeling approach to discover novel inhibitors of SHP2. A detailed schematic overview of the methodology employed summarizes the workflow. The results of this work are explained in detail in the following sections (Figure 2).

### 2.1. Generation of Receptor-Based Pharmacophore Model

**Figure 3:**
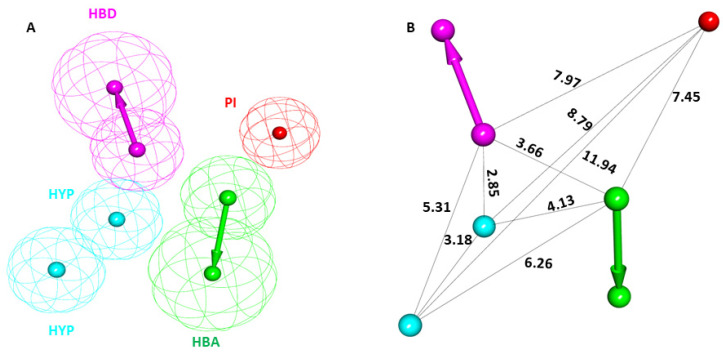
The chemical characterization and interfeature distance of the selected pharmacophore model. (**A**) Magenta, cyan, green, and red colors represent hydrogen bond donor (HBD), hydrophobic (HYP), hydrogen bond acceptor (HBA), and positive ionizable (PI) features, respectively. (**B**) The interfeature distance of the selected model is displayed in Å.

The SHP099-bound crystal structure of the SHP2 E76D protein (PDB: 6CMR) was obtained from the PDB database. The literature suggests that the SHP099 inhibitor can limit cancers with the SHP2 E76D mutation, making this complex a promising candidate for pharmacophore modeling [19]. The SHP099-SHP2 complex was imported to DS and subsequently submitted for the pharmacophore model generations using the module *Receptor–Ligand Pharmacophore Generation* in the Pharmacophore section of DS. A total of ten pharmacophore models were generated. Out of the ten, the top-ranked pharmacophore model with the highest selectivity score was selected as a potential candidate (Table S1). The selected PM displayed a selectivity score of 10.99 generated from the SHP2-SHP099 complex. The initially selected PM displayed five different features: one hydrogen bond donor (HBD), one hydrogen bond acceptor (HBA), two were hydrophobic (HYP), and one was positive ionizable (PI). The prepared PM and the respective interfeature distance are shown in Figure 3A,B.

### 2.2. Pharmacophore Model Validation

The *Güner–Henry* (GH) and *Enrichment factor* (EF) approach was used to validate the selected pharmacophore model to evaluate its efficiency [20,21]. The GH method, also known as the goodness of hit list, is the linear combination of two dependent variables, the percent yield of actives and the percent ratio of the actives in the hit list (Table 1). The EF indicates the enrichment of the hit list concerning the database. A decoy dataset was compiled using 250 inactive (IC_50_ ≥ 100 nm) and 20 active (IC_50_ ≤ 100 nm) compounds. Subsequently, the prepared decoy dataset was screened on Hypo1 using the *Ligand Pharmacophore Mapping* module of DS to validate the pharmacophore. The mapping results demonstrated that Hypo1 effectively mapped 95% of active compounds with an acceptable GH score of 0.81 and EF value of 10.68. To be an ideally acceptable model, the pharmacophore model must have a GH score above 0.60 [21,22]. Other parameters employed for pharmacophore validation, such as the percentage yield of actives, percentage ratio of actives, false positives, and false negatives, are included in the decoy dataset (Table 1). The validation results strongly suggested that Hypo1 can efficiently differentiate between active and inactive compounds against SHP2 and, therefore, can be utilized for further virtual screening.

**Figure 4:**
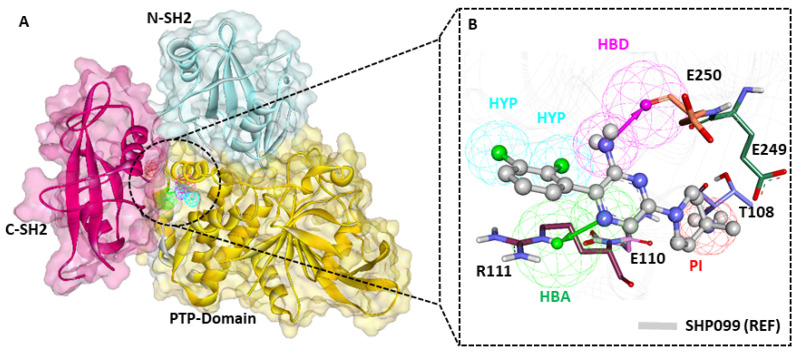
Receptor–ligand-based pharmacophore model. (**A**) Detailed domain structure of SHP2 PDB: 6CMR, allosteric sites are shown in blue and red colors. (**B**) The final pharmacophore model mapped with co-crystalized drug SHP099 (REF) is shown in a gray color with a ball-and-stick model. The key residues R111 and E250 were mapped with hydrogen bond acceptor and donor features, respectively.

The pharmacophore model (PM) overlay on the SHP2–inhibitor complex indicates that the PM effectively maps the key residues of the binding pocket (Figure 4A). The detailed insights reveal that the dichloro substituents from the chlorophenyl ring of SHP099 (REF) mapped to the HYP feature. The amine group present in the pyrazin ring mapped to the HBD feature, the nitrogen group present in the pyrazin ring mapped to the HBA feature, and the amino group substituted at the fourth position of the piperidin ring accommodated to the PI feature (Figure 4B). The protein residue mapping site of the pharmacophore model aligns with binding site residues such as R111 and E250, via bond acceptor (HBA) and hydrogen bond donor (HBD) features, respectively. Notably, these residues have been reported as crucial for substrate recognition and SHP2 activation. Based on the pharmacophore model’s alignment with allosteric site residues of SHP2, we believe drug-like compounds matching these highlighted residues could target SHP2 with high specificity and selectivity (Figure 4B).

### 2.3. Virtual Screening

For the pharmacophore-based virtual screening, we used four different chemical databases including ZINC natural (144,766), Eximed (86,640), InterBioScreen natural (505,304), and Marvin supernatural (325,319). The prepared databases were filtered based on their physicochemical and pharmacokinetic properties by applying Lipinski’s Rule of Five (Ro5) and the ADMET descriptors module available in DS (Table S2). In the ADMET descriptors, specific values such as level 0 for absorption indicate good intestinal absorption, solubility level 3 indicates good solubility, and a blood–brain barrier (BBB) level of 3 was set to ensure low penetration into brain cells. After applying the Ro5 and ADMET descriptors filters, we identified 31,277 compounds suitable for further pharmacophore-based screening. The Ligand Pharmacophore Mapping protocol (LPM) available in DS was used to screen the obtained compounds. SHP099 (REF), showing a fit value of 4.43, was applied as a criterion to reduce the resulting compounds further. In ligand pharmacophore mapping, the “Fit value” refers to a numerical score that quantifies how well a particular ligand fits into the pharmacophore features of the selected hypothesis. A total of 6536 compounds were successively mapped to Hypo1 through LPM. As a result, 518 compounds with a fit value higher than REF (fit value > 4.43) were chosen for the molecular docking study.

### 2.4. Molecular Docking

The 3D structure of the SHP2 protein with the E76D mutant (PDB ID: 6CMR) was taken as a receptor for the docking study [19]. The structure was prepared using DS by deleting the heteroatoms. Subsequently, the binding site was defined using the *Define and Edit Binding Site* module available in DS (*v2023*) within the cavity of bound inhibitor SHP099 (REF). The XYZ coordinates were set as −1.38, 12.93, and 21.13, and the radius of the identified docking sphere was defined within 8 Å. For the molecular docking of compounds obtained through pharmacophore-based virtual screening, we used *Genetic Optimization of Ligand Docking* (*GOLDv5.2.2*). Docking validation was conducted by redocking REF (SHP099) in the same cavity before actual MD studies (Figure S1). The selective inhibitor REF was also docked during the MD experiment, and 518 compounds were used using the SHP2 receptor. The final hit compounds were sorted based on default docking scores (GoldScore and ChemScore) provided in GOLD. The results of our MD study demonstrated that REF displayed a score of 55.33 and Chemscore −28.35. Therefore, compounds having a docking score greater than REF were selected as potential SHP2 binders, and based on the compound’s detailed molecular interactions with the key residues for inhibiting SHP2 allosterically, a total of 71 compounds were shortlisted. We further analyzed each. Fifteen compounds showing hydrogen bond interaction with R111, E250, and other key residues were selected for further calculation. The 2D representation of corresponding molecules and their MD scores are shown in Table S3.

### 2.5. Molecular Dynamics Simulation

Molecular dynamic simulations (MDSs) provide insights into the dynamic behavior of molecular systems, such as proteins, nucleic acids, and small molecules, at atomic-level detail [23]. Initially, a 50 ns MDS run was performed for all 15 selected complexes obtained from the MD experiment to save time and expenses. The stability analysis of the simulated trajectories was studied using root-mean-square deviation (RMSD) and root-mean-square fluctuations (RMSFs). The potential ligands were ranked based on their binding free energy calculated using MM-PBSA in the next step. The potential five simulation systems were further selected and prepared using the GROMACS program for simulation run until 500 ns. The final three hits (Lig_1, Lig_6, and Lig_14) were chosen based on stability, binding affinity, and key molecular interactions.

#### 2.5.1. Stability Assessment of Simulated Complexes

**Figure 5:**
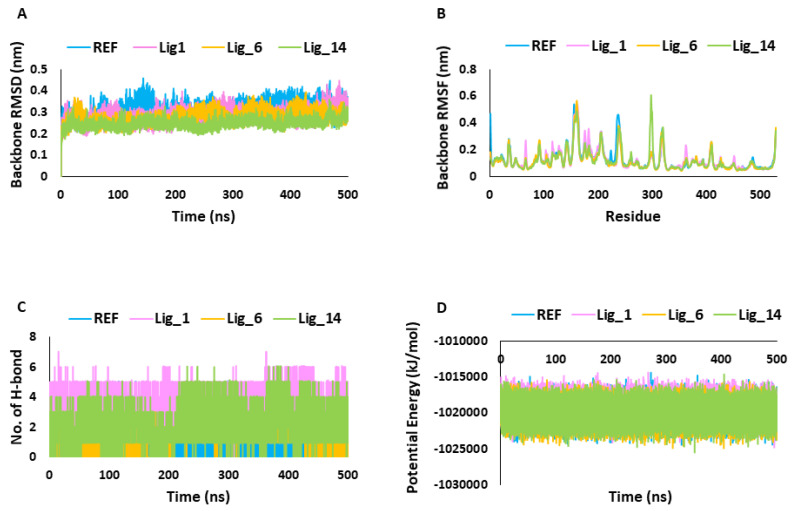
Molecular dynamic simulation analysis. (**A**,**B**) Backbone RMSD and RMSF. (**C**) Number of H-bonds. (**D**) Potential energy.

The MDS results were analyzed through RMSD, RMSF, potential energy, hydrogen bond analysis, and binding mode analysis. MDS trajectories were used to analyze the system stability throughout the simulation run. Over the period of the 500 ns simulation run, the RMSD values of the identified hits and REF were observed within the range of threshold fluctuation of <0.3 nm. Interestingly, Lig_14 displayed significantly low RMSD values of 0.24 nm, followed by Lig_6 at 0.27 nm, Lig_1 at 0.28, and REF at 0.31 nm which suggests that our identified hits showed stable behavior throughout the simulation run compared to REF (Figure 5A). The root-mean-square fluctuation (RMSF) signifies the protein’s residual flexibility during the simulation run. In the present work, the RMSFs of the SHP2 backbone atoms were observed for 500 ns, and we observed that the major fluctuations occurred in the C-SH2 domain, ranging between residue numbers 112 and 216. The other domain responsible for atomic fluctuations was the PTP domain (221–525aa). We observed the overall RMSF average values, and they were <0.3 nm in all the simulated complexes, indicating the stability of the systems (Figure 5B). The hydrogen bond analysis was performed along with other MDS calculations to obtain a more comprehensive understanding of each system’s dynamics and function. The hydrogen bond analysis of selected simulated complexes revealed that identified hits form a more prominent and greater number of hydrogen bonds than REF (Figure 5C) and can bind to the receptor more tightly. Further, the potential energy plots suggested that the identified hits showed steady behavior during the 500 ns MDS run compared to REF (Figure 5D and Table 2).

#### 2.5.2. MM-PBSA Calculation

**Figure 6:**
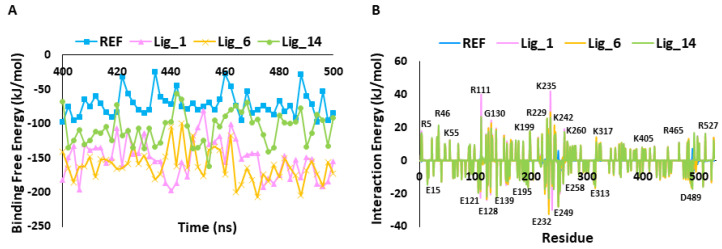
(**A**) Calculation of binding free energy for REF and identified hit molecules Lig_1, Lig_6, and Lig_14, calculated using MM-PBSA method. (**B**) Per residue energy decomposition of REF and hits.

The binding affinity of the hit candidates for the SHP2 receptor was inferred by calculating the binding free energy (ΔG) using the *MM-PBSA* method. The last 100 ns trajectory files were used for calculating the ΔG values. The observed average binding free energy value was for Lig_1 −161.49 kJ/mol, Lig_6 −151.28 kJ/mol, Lig_14 −107.13 kJ/mol, and for REF, a binding free energy of −71.48 kJ/mol was observed (Figure 6A). The ΔG values emphasized that the identified hits have a greater binding affinity towards SHP2 than REF.

The per-residue contribution is an important approach for understanding detailed protein inhibitor interactions and each residue’s role in the macromolecule’s stability. Figure 6B shows that the REF inhibitor (SHP099) and selected hits target similar residues, but their energetics differ. In particular, E15, E121, E128, E139, E195, E232, E249, E258, E313, and D489 are the key contributors to ligand binding via forming hydrophobic interactions. On the other hand, residues placed on the upper part of the graph, such as R5, R46, K55, R111, G130, K199, R229, K235, K242, K260, K317, K405, R465, and R527, contribute to polar interactions.

#### 2.5.3. Principal Component Analysis

**Figure 7:**
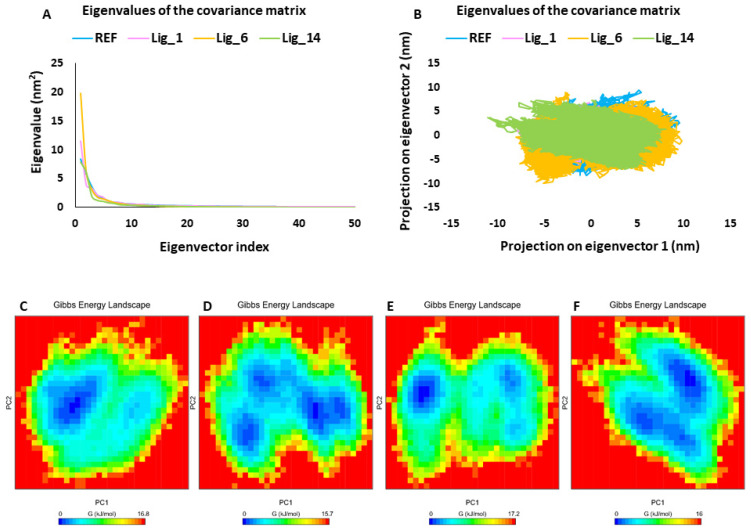
PCA. (**A**) The eigenvector index, (**B**) PC1 and PC2, and (**C**–**F**) free energy landscape of REF, Lig_1, Lig_6, and Lig_14, respectively. Blue spots in the plots indicate the energy minima, whereas the red color represents a higher energy conformation.

Principal component analysis (PCA) is commonly used to investigate the coordinated movements in protein–ligand complexes by focusing on the c-alpha atoms [24,25]. The PCA of the selected SHP2–ligand complexes revealed that the first few eigenvectors play a crucial role in the overall motion. Figure 7A displays the superimposed plot of the first 50 eigenvectors from the SHP2-REF, SHP2-Lig_1, SHP2-Lig_6, and SHP2-Lig_14 complexes. The comparative analysis indicated that the SHP2-Lig_6 complex might exhibit greater conformational variability, whereas the SHP2-Lig_14 complex displayed the least motions compared to the other complexes. Additionally, we examined the dynamics of protein–ligand systems through 2D projection plots of the first two PC1 and PC2 prime contributing eigenvectors. The overlay of these plots showed that all hit compound-bound complexes were located in a region similar to that of the SHP2-REF complex (Figure 7B). Consistent with the eigenvector analysis, Lig_14 exhibited the most stable clustering; this was followed by REF, Lig_1, and Lig_6, respectively. The analysis of the GFE landscape based on PC1 and PC2 revealed that energy values spanned from 0 to 16.8 kJ/mol for REF, 0 to 15.7 kJ/mol for Lig_1, 0 to 17.2 kJ/mol for Lig_6, and 0 to 16 kJ/mol for Lig_14. The energy values indicated that Lig_1 and Lig_14 displayed lower free energy values than REF, suggesting that these complexes might be thermodynamically more stable. Lig_6 indicated slightly higher energy values than REF and a lower minimum energy state, as shown in blue (Figure 7C–F). Collectively, PCA and GFE landscape energy analysis revealed that the selected hit compounds displayed comparable or better results than the REF inhibitor and, therefore, can act as a good candidate against the SHP2 inhibition program.

### 2.6. Binding Mode Analysis

**Figure 8:**
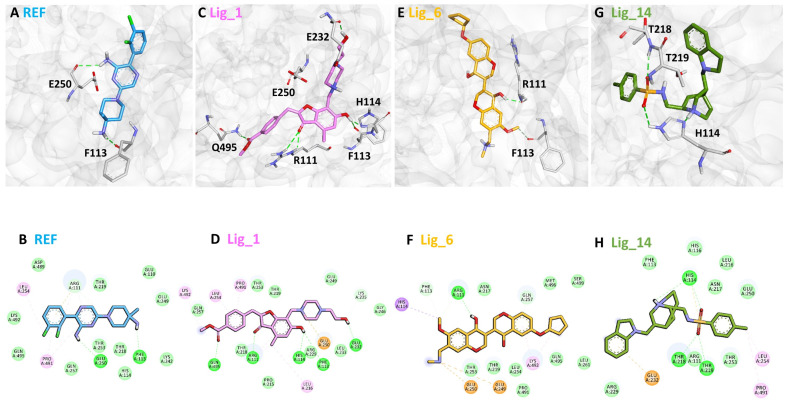
The binding modes of (**A**) REF, (**C**) LIG_1, (**E**) Lig_6, and (**G**) Lig_14 with the allosteric site residues of SHP2. REF, Lig_1, Lig_6, and Lig_14 are shown in blue, pink, yellow, and green, respectively, represented in the stick model. The lower panel represents the 2D molecular interaction of (**B**) REF, (**D**) Lig_1, (**F**) Lig_6, and (**H**) Lig_14 with active site residues. The hydrogen bonds are shown in green dashed lines, while π-π, π-alkyl, π-cation π-sulfur, and π-σ interactions are shown as pink, orange, yellow, and purple dashed lines, respectively.

The binding affinity of the selected hits for the SHP2 allosteric site was analyzed using the average complex obtained from the last 5 ns MDS trajectories (Figure S2). In superimposing the protein–ligand complexes, we observed that the identified hit compounds have a similar binding mode to the REF inhibitor SHP099 (Figure 8). The allosteric site1 of the SHP2 protein is located at the C-SH2-PTP interface, contributing to the stability of the auto-inhibited conformation of SHP2. Previous work conducted on SHP2 suggested that E110 present at allosteric site1, R265 at allosteric site2, and R501 located at the Q loop are the key residues that participate in the stabilization of the auto-inhibited conformation of SHP2 [26]. On the other hand, R111 is involved in substrate recognition; it can interact by forming an H-bond, an electrostatic bond with the target that inhibits phosphatase activity, depending on the inhibitors. The SHP2-SHP099 co-crystalized structure (PDB: 6CMR) shows the H-bond interaction with T108, E110, R111, E249, and E250 [19]. By analyzing the molecular interaction pattern of the docked pose of REF with SHP2 after 500ns MDS, it was observed that REF is making hydrogen bond interaction with the residues P113 and E250 of the target protein (Figure 8A,B). Based on REF molecular interaction, we selected only those hits that target T108, E110, R111, E249, and E250 via hydrogen preferably or hydrophobic interactions.

The detailed analysis of the binding mode explains that the average structure of the SHP2-Lig_1 complex displayed five hydrogen bond interactions. The benzofuran ring of Lig_1 forms hydrogen bonds with residues R111, F113, and H114 (Table 3). Residue E232 forms a hydrogen bond with the piperazine ring of Lig_1 and Q495, making hydrogen bond interactions with the methyl benzoate ring (Figure 8C). Additionally, E250 stabilizes the protein by forming a π–cation interaction. Lig_1 displayed significant π–alkyl interactions with residues L216, E250, L254, K492, and P491 (Figure 8D). Molecular interactions were also supported via various van der Waals interactions with E110, H114, T219, K242, E249, D489, K492, and Q495 (Table 3). The hit, Lig_6, displayed one hydrogen bond with residue R111 (Figure 8E). F113, N217, T219, T253, L254, Q257, L261, P491, Q495, and M499 contribute to van der Waal interactions (Figure 8F). The π–alkyl interactions were observed with residues similar to Hit1. Similarly, the benzene sulfonamide ring of Lig_14 forms three hydrogen bonds with the residues H114, T218, and T219 (Figure 8G). It was also observed that the residue H114 makes the protein–ligand complex stable by forming a salt bridge interaction. Residues L254 and P491 form π–alkyl interactions, whereas R111, F113, H116, L216, N217, R229, E250, and T253 are responsible for making van der Waals interactions (Figure 8H). The binding pattern of the identified hits and REF are shown in (Figure S2). The 2D structure of identified hits along with the SMILE ID and the IUPAC name are mentioned in Table 4.

### 2.7. Pharmacokinetic Property Assessment

Assessments of the pharmacological properties of the absorption, distribution, metabolism, and excretion (ADME) of a chemical compound are crucial steps for their selection as potential lead candidates. The available computational tools and web servers allow researchers to predict the ADME properties of identified compounds in the initial stage of drug discovery and save a lot of time and expenditure for being invested in compounds with unfavorable properties [27].

The present study’s results suggested that the identified hits have better water solubility and are compatible with REF. The *caco-2* permeability prediction is a useful parameter for the oral absorption of a drug. The benchmark for a drug to be absorbed at the intestinal level is >30%. Those that have intestinal absorbance levels <30 are considered poorly soluble and less absorbed. The skin permeability potential score is higher than the acceptable range for REF. In the next step, the hits and REF were investigated for being a p-glycoprotein substrate and inhibitor, and we observed that all three compounds were predicted as a p-glycoprotein substrate. A drug considered a substrate of *p-glycoprotein* can potentially act as an inhibitor or inducer of its function; p-glycoprotein functions as a biological barrier by removing xenobiotics and toxins from the cell [22]. The volume of distribution (VD) measures the relationship between the administered dose of a drug and the amount of drug present in plasma to tissue; a higher value of the VD indicates that more of a drug is distributed in tissue. REF and hits were observed to be fairly distributed in tissue. The pharmacokinetic parameters, blood–brain barrier permeability (BBBP), and central nervous system permeability (CNSP) for REF and hits were analyzed, and it was concluded that all the compounds have a very rare chance of causing CNS-related toxicity. The pharmacokinetic parameter that analyzes the metabolism of a compound includes cytochrome P450, an important enzyme in the human body for drug detoxification. In our work, we considered all the isoforms of cytochrome P450; the overall calculation emphasized that all three hits have acceptable results compared to REF. The combination of hepatic and renal clearance encounters drug clearance from the body. The excretion of the drug is also measured by Organic Cation Transporter 2 (OCT2), which is unambiguously expressed on the tubular epithelia of the kidney and plays a critical role in drug renal clearance (Table 5). We observed that REF and the hits do not inhibit hERG I (the human ether-à-go-go-related gene), an important indication for a compound not causing cardiotoxicity when administered. On the other hand, similar to REF, all three identified hits inhibit *hERG II*. The Oral Rat Acute Toxicity (LD_50_) and Chronic Toxicity (LOAEL) were also predicted for Lig_1, Lig_6, Lig_14, and REF. Other parameters like *AMES toxicity,* hepatotoxicity, skin sensitization, *Minnow* toxicity, and *T. Pyriformis* toxicity were also predicted for REF and hit (Table 6). The overall analysis of the ADMET calculation strongly suggested that the identified hits Lig_1, Lig_6, and Lig_14 show predicted values in the acceptable range compared to REF.

## 3. Discussion

The non-receptor tyrosine phosphatase SHP2 is a key player in various cell signaling pathways, and nowadays, it is being explored as an oncogene in many tumors which makes SHP2 an interesting therapeutic target [6]. SHP2 is involved in the downstream signal transduction of multiple growth factors, and its requirement for complete RAS-RAF activation and its role in the negative regulation of the JAK-STAT pathway have established SHP2 as a key contributor in oncogenic signaling pathways [28]. Ruess et al. reported the established role of SHP2 in oncogenic KRAS-driven tumors and provided the necessary support for proving the role of SHP2 in the positive regulation of KRAS-driven carcinogenesis [28]. Several recent efforts have been made to find potential selective SHP2 inhibitors. Some inhibitors have reached clinical trials, but none have been FDA-approved to date [17,19,29]. Considering the importance of the SHP2 receptor in cell proliferation and growth, there is a requirement to develop a selective SHP2 inhibitor that can be a potential candidate for clinical trials. The selection and development of novel drug candidates against the macromolecule are complex and time-consuming. To potentiate this drug development process, we can use computer-aided drug-designing methods [30].

The present study focuses on the structure-based pharmacophore modeling approach, one of the most promising in silico techniques for drug design (Figure 3 and Figure 4), which was selected along with other validation methods, such as MD and MDS (Figure 5 and Figure 6). Based on the previous studies conducted on SHP2, we selected 6CMR for PM; a total of 10 PMs were produced using the *Receptor–Ligand Pharmacophore Generation* protocol of DS (Table S1). The pharmacophore mapping features SHP2 allosteric site residues and a 6CMR-bound ligand SHP099 map with T108, E110, R111, E249, and E250 (Figure 4B). The generated PM had five chemical features: one HBA, one HBD, two HYP, and one positive ionizable (P) feature [28]. The HBA feature could map E110 and R111, and the HBD feature was mapped with E250, whereas the P feature was mapped with T108. In the present investigation, the pharmacophore feature mapping results suggest that the selected PM can be a significant candidate for the virtual screening of chemical databases. The PM was subsequently validated using the GH approach (Table 1) [21]. A drug-like database was prepared from four chemical databases utilizing ROF and ADMET descriptor filters available DS using the selected PM (Table S2). Out of 31,277 compounds, only 518 were mapped to the selected PM. The reduction of millions of compounds to hundreds reveals the significance of the pharmacophore-based virtual screening process.

The final 518 compounds were then subjected to the GOLD program for MD analysis [31]. For comparative analysis, the 6CMR co-crystal ligand SHP099 was docked under similar conditions [3,32]. Therefore, the SHP099 (REF) docking score was used as the first criterion for choosing the compounds. The selected REF pose has a Goldscore of 55.28 and a Chemscore of −28.35. In this work, the final selected compounds through MD were further subjected to MDS using the GROMACS program [33]. The simulated protein–ligand complexes were ranked based on BFE, and the compounds with better binding affinity for SHP2 than REF were selected (Table 2). The selected hits were analyzed based on their stability using geometrical parameters like RMSD and RMSF (Figure 5A,B).

The results obtained in the present work reveal that all three identified hits showed stable behavior through a 500 ns MDS run, and the observed average threshold value was <0.3 nm. The identified hit also displayed strong hydrogen bond-forming ability, suggesting that these hits have great affinity for SHP2 (Figure 5C). The MM-PBSA method was used to validate the binding affinity of complexes (Figure 7A and Table 2) [34]. The average binding free energy from the last 100 ns trajectories revealed that all three identified hits, Lig_1, Lig_6, and Lig_14, displayed a significantly better binding affinity with values of −151.13 kJ/mol, −161.49 kJ/mol, and −107.13 kJ/mol, respectively, when compared with REF −71.48 kJ/mol. It can be noticed that REF and the selected hits target similar residues with different energetics. In particular, E15, E121, E128, E139, E195, E232, E249, E258, E313, and D489 significantly contribute to binding via various hydrophobic interactions. The residues shown on the upper side of the graph, such as R5, R46, K55, R111, G130, K199, R229, K235, K242, K260, K317, K405, R465, and R527, may contribute to polar interactions. (Figure 6B). The detailed binding mode of the hit compounds and SHP099 showed that allosteric site key residues were targeted by various types of molecular interactions (Figure 8 and Table 3).

In addition, PCA was used to study the collective motion of the selected protein-ligand complexes [25]. The eigenvector index revealed the significance of the first five eigenvectors which were regulating the overall motion of the protein. Moreover, the covariance analysis revealed that all three hits (Lig_1, Lig_6, and Lig_14) occupied less conformational space than REF (Figure 7A,B). Gibb’s free energy (GFE) landscapes are useful measures for studying the thermodynamic stability of the compounds [35]. The analysis of the GFE landscape based on PC1 and PC2 revealed that energy values spanned from 0 to 16.8 kJ/mol for the REF, 0 to 15.7 kJ/mol for Lig_1, 0 to 17.2 kJ/mol for Lig_6, and 0 to 16 kJ/mol for Lig_14. The energy values indicate that Lig_1 and Lig_14 displayed lower free energy values than REF, suggesting that these complexes might be thermodynamically more stable. Based on all computational calculations, we propose that the identified hits can serve as a potential scaffold for developing anticancer agents.

The pharmacoinformatic techniques used in the present study provide significant results, but our work still has some limitations. Firstly, for pharmacophore modeling, we used a single protein-ligand complex (PDB: 6CMR), which can limit the conformation flexibility of SHP2. To overcome this issue, we used the long MD simulation run. For efficient pharmacophore-based virtual screening, we consistently used the REF molecule in each step to increase the credibility of the methodology employed.

## 4. Materials and Methods

### 4.1. Generation of Receptor-Based Pharmacophore Model

The 3D structure of the SHP2 mutant E76D protein in complex with the selective allosteric selective1 inhibitor SHP099 was downloaded from the RCSB Protein Data Bank with PDB code 6CMR having a resolution of 2.21 Å [19]. The obtained protein structure was further cleaned and prepared using the ‘clean protein’ and ‘prepare protein’ protocols in Discovery Studio v23, respectively. The pharmacophore model (PM) was generated using the Receptor–Ligand Pharmacophore Generation tool available in DS [36]. The pharmacophore generation module embedded in DS first identifies the features that match the receptor–ligand interactions, and based on those features, it generates 10 pharmacophore models.

### 4.2. Pharmacophore Model Validation

The prepared pharmacophore was validated using a well-known Güner–Henry (GH) approach [20,21,22]. The validation of the PM is a crucial step for identifying the ability of selected models to identify and differentiate between given active and inactive compounds [20,21]. In this study, the Ligand Pharmacophore Mapping protocol, incorporating a flexible search option within Discovery Studio (DS), was employed to screen the decoy test set. The results obtained from ligand pharmacophore mapping were subsequently utilized to validate the prepared pharmacophore model by calculating the GH score and EF value using the specified equations.

$$GH=\left\{[\frac{Ha\left[3A+Ht\right]}{4HtA}]\left[1-\frac{Ht-Ha}{D-A}\right]\right\}$$

EF=(Ha×D)/(Ht×A)

where, D represents the total number of molecules in the database, A is the number of active molecules, Ht is the total number of active molecules in the retrieved hits, and Ha represents the number of retrieved hits by pharmacophore. The % yield of actives is shown by (Ha/Ht) × 100, and the % ratio of actives is represented by Ha/A × 100. Meanwhile, A-Ha and Ht-Ha are the denotations for false negative and false positive specified equations.

### 4.3. Virtual Screening

Four chemical databases (Eximed, ZINC natural, Marvin supernatural, and InterBioScreen) were selected to identify potential SHP2 allosteric inhibitors. To identify compounds with drug-like properties from the chemical databases containing thousands of compounds, we initially applied the ADMET descriptors and the drug-likeness rule known as Lipinski’s Rule of Five (Ro5), available in Discovery Studio (DS). This screening aimed to select compounds with acceptable pharmacokinetic characteristics [22,37,38]. In the next step, the selected validated PM was subjected as an input query to screen out the prepared drug-like database using Ligand Pharmacophore Mapping with the Best mapping option of DS [36].

### 4.4. Molecular Docking

In this work, the *Genetic Optimization of Ligand Docking* (GOLD v5.2.2) was considered for the molecular docking calculation [39]. The crystal structure of the human SHP2 receptor, bound with known inhibitor SHP099 (PDB ID: 6CMR), was downloaded from the Protein Data Bank (http://www.rcsb.org/) accessed on 20 December 2022. Before performing molecular docking studies, the protein was prepared, all missing atoms were added, and bond orders were corrected using the Clean Protein module, available in DS [40]. The energy minimization of the protein was conducted using a CHARMm27 force field. The binding site of the SHP2 receptor was specified around the bound allosteric inhibitor SHP099. The molecular docking studies using the GOLD Genetic Algorithm (GA) generated a maximum of ten poses for each drug-like molecule subjected to it. Goldscore and Chemscore are the default scoring functions to select potential SHP2 binders. The bound inhibitor SHP099 (REF) was considered the reference criterion for analyzing the docking results. Compounds that displayed better docking scores and optimal binding modes with the mentioned key residues were used for SHP2 inhibition. The results were visualized in DS and compared with the reference inhibitor for further study.

### 4.5. Molecular Dynamics Simulation

The compounds secured through the molecular docking analysis were further passed on to MDS using the *Groningen Machine for Chemical Simulations* (GROMACS v5.15) to analyze the physical atomistic movements of molecules under the given virtual physiological condition [33]. The protein parameter and coordinate files were generated by using the CHARMm27 force field [41]. Topology files for the ligands were generated with *SwissParam* [42]. A dodecahedron box and TIP3P water model were used for each simulation system. The prepared SHP2–ligand simulation systems were further neutralized by adding appropriate Na^+^/Cl^−^ ions. The *Steepest Descent* algorithm was used to minimize the steric hindrance of each system before running the actual MDS. The equilibration of protein–ligand complex systems was conducted by incorporating NVT and NPT ensembles for 1 ns at 300 K. The MDSs were performed under periodic boundary conditions to avoid edge effects for 500 ns of the simulation run. The *Leap-Frog* and *LINC* algorithms were used for non-bonding interactions and to restrain the bond length. *Particle mesh Ewald* (PME) was employed to estimate long-range electrostatic interactions. The MDS output was analyzed using the DS and GROMACS trajectory analysis tools.

#### 4.5.1. Stability of Simulation Systems

To evaluate simulation systems’ stability, the protein’s dynamics upon ligand binding were quantitatively assessed using root-mean-square deviation (RMSD), root-mean-square fluctuations (RMSFs), and potential energy calculations. The calculations were carried out utilizing the “*gmx rmsd*”, “*gmx rmsf*”, and “*gmx energy*” commands in GROMACS.

The RMSD calculation was performed using the following equation.

$${RMSD}_{x}=\sqrt{\frac{I}{N}\sum_{i=1}^{N}{\left(r^{\prime }\left(t_{x}\right)\right)-(r_{i}(t_{ref}))}^{2}}$$

where the number of atoms is N, *t_ref_* is the reference time, *r*′ represents the location of selected atoms within the frame *x* after superimposition on the reference frame, and the recoding intervals of *x* are designated with *t_x_* [22].

Additionally, hydrogen bond interactions between the protein and ligand were analyzed for each system, employing the “*gmx hbond*” command to identify and quantify hydrogen bonds, providing insight into the complexes’ binding affinity and structural integrity.

#### 4.5.2. MM-PBSA Binding Free Energy Calculation

In computational drug discovery, the binding free energy of a system can provide a mechanistic insight into protein–ligand interaction, which can serve as a crucial measure to calculate the absolute binding energy of identified hits. This study used the “*g_mmpbsa*,” a GROMACS plugin tool, to calculate binding free energy [34]. From the 500 ns MDS trajectories, the last 100 ns trajectories were used to calculate binding free energy. The ΔG of the protein–ligand complex was calculated by using the following equation:

$$G_{binding}=G_{complex}-\left[G_{protein}+G_{ligand}\right]$$

G_binding_ is the binding free energy of a protein–ligand complex, G_complex_ represents the total energy of the protein–ligand complex, and G_protein_ and Gl_igand_ denote individual energy components.

#### 4.5.3. Principal Component and Free Energy Landscape Analysis

The fluctuations in protein residues on protein–ligand binding were observed through principal component analysis (PCA) using simulated MDS trajectories [43]. Also, Gibbs free energy (GFE) landscape values were calculated for the thermodynamic properties of protein–ligand complexes [35]. The GROMACS tool “*gmx_covar*” was used to analyze pattern recognition in protein movements [23]. In PCA, the covariance matrix calculates the eigenvectors and eigenvalues [44]. The larger the eigenvalue of the corresponding eigenvector, the higher the motion for this eigenvector coordinate [45]. The “*gmx_anaeig*” tool was used to produce the 2D plot of two different eigenvectors, and subsequently, the “*gmx_sham*” GROMACS tool was exploited to calculate the GFE landscape for these components [23]. PC1 and PC2 were used to calculate the GFE based on the following equation:

$$\Delta G\left(PC1,\;PC2\right)=-KBTlnP\;(PC1,\;PC2)$$

PC1 and PC2 are reaction coordinates, KB symbolizes the Boltzmann constant, and P (PC1, PC2) illustrates the probability distribution of the system along the first two principal components [46].

The change in Gibbs free energy (ΔG) for a system depends upon the change in enthalpy (ΔH) and the change in entropy (ΔS) according to the following equation [47]:

(1) ΔG=ΔH−TΔS

where ΔH = enthalpy, T = temperature in Kelvin, ΔS = entropy, and ΔG = Gibbs free energy.

### 4.6. Pharmacokinetic Properties Analysis

The process of drug discovery is challenging and time-consuming. Bringing a new pharmaceutical drug into the market takes several years. Investigating a potential compound’s pharmacokinetic (ADME) properties is crucial before being upgraded to clinical trials to avoid failure and save time. In the present study, selected hit compounds were submitted to the online server pkCSM (http://structure.bioc.cam.ac.uk/pkcsm) to analyze their detailed pharmacokinetic or ADMET properties accessed on 2 January 2024 [27].

## 5. Conclusions

The present research aimed to find a selective SHP2 allosteric inhibitor. A structure-based pharmacophore model (PM) was generated using the known SHP2 inhibitor-bound crystal structures (PDB: 6CMR). The PM was observed to efficiently target the key residues of the allosteric site1 residue through desirable hydrogen bond interactions. Pharmacophore-based virtual screening was employed to obtain potential SHP2 inhibitors from the prepared drug-like database. The MD and MDS analysis were used to predict and validate the binding affinity of the compounds. In the next step, the binding free energy and Gibb’s free energy were used to rank the simulated complexes, and the results were compared with the bound selective allosteric inhibitor SHP099 (REF). The detailed molecular interaction analysis further revealed that the selected hits favorably interact with SHP2 by forming more hydrogen bonds or electrostatic interaction with the key residues. As a result, we propose that the identified hits might be a strong contender against SHP2 for anticancer therapies. To overcome the limitations of computational drug discovery, we used the REF drug as a control, and the results of the identified hits were compared with REF in each step. In conclusion, we strongly suggest that with our computational results, further experimental validation can be a good measure to prove the potentiality of the identified hits.

## Figures and Tables

**Table 1:** Pharmacophore validation result from GH method using decoy test set.

Sr. No.	Parameters	Calculated Values
1	Total no. of molecules in the database (D)	270
2	Total number of active molecules in the database (A)	20
3	Total number of active molecules in the retrieved hits (Ht)	24
4	Number of retrieved hits by pharmacophore (Ha)	19
5	% Yield of actives [(Ha/Ht) × 100]	79.16%
6	% Ratio of actives [(Ha/A × 100)]	95%
7	False negative [A-Ha]	1
8	False positive [Ht-Ha]	5
9	Goodness of fit	0.81
10	Enrichment factor (EF)	10.68

**Table 2:** The details of molecular docking and molecular dynamics simulation analysis after 500 ns of the selected hit compounds.

Systems	Docking Score		RMSD (nm)	RMSF (nm)	Potential Energy (kJ/mol)	Number of Hydrogen Bonds	Binding Free Energy (ΔG_binding_ kJ/mol)
	Goldscore	Chemscore	Backbone Atoms	Backbone Atoms			
REF	55.28	−28.35	0.31	0.12	−1,019,870	1.53	−71.48
Lig_1	79.37	−37.47	0.28	0.12	−1,019,239	3.57	−151.28
Lig_6	73.16	−20.03	0.27	0.11	−1,020,092	1.08	−161.49
Lig_14	68.37	−26.43	0.24	0.12	−1,019,950	2.49	−107.13

**Table 3:** The detailed binding mode analysis of the identified hits and REF.

Name	Hydrogen Bond Interactions				van der Waals Interactions	π-π/π–Alkyl Interactions
	Amino Acid	Amino Acid Atom	Ligand Atom	Distance (<3.5 Å)		
Lig_1	Arg111	HE	O1	2.13	Glu110, His114, Thr219, Lys242, Glu249, Asp489, Lys492, Gln495	Leu216, Glu250, Leu254, Lys492, Pro491
	Phe113	O	H43	2.76		
	His114	ND1	H43	1.93		
	Glu232	O	H62	1.80		
	Gln495	HE21	O28	2.18		
Lig_6	Arg111	O	H41	2.05	Phe113, Asn217, Thr219, Thr253, Leu254, Gln257, Leu261, Pro491, Gln495, Met499	His114, Glu249, Glu250, Lys492
Lig_14	His114	HE2	O9	2.89	Arg111, Phe113, His116, Leu216, Asn217, Arg229, Glu250, Thr253	Glu232, Leu254, Pro491
	Thr219	NH	O10	2.37		
REF	Phe113	O	H42	2.06	Glu110, His114, Thr219, Lys292, Glu249, Asp489, Lys492, Gln495	Leu254, Pro491
	Glu250	O	H37	2.68		

**Table 4:** IUPAC names SMILE code and 2D structure of identified hits Lig_1, Lig_6, and Lig_14.

Characters	Lig_1	Lig_6	Lig_14
IUPAC name	methyl 4-[[6-hydroxy-7-[[4-(2-hydroxyethyl)piperazin-1-ium-1-yl]methyl]-4-methyl-3-oxo-benzofuran-2-yl]methyl]benzoate	[(3R,4R)-3-[(3S,4S)-6-(cyclopentoxy)-4-hydroxy-chroman-3-yl]-4-hydroxy-6-methoxy-chroman-7-yl]methyl-methyl-ammonium	N-[[(1S,2R,4S,5S)-5-(indolin-1-ylmethyl)quinuclidin-1-ium-2-yl]methyl]-4-methyl-benzenesulfonamide
SMILE ID	COC(=O)c1ccc(CC2Oc3c(C[NH+]4CCN(CCO)CC4)c(O)cc(C)c3C2=O)cc1	C[NH2]Cc1cc2OC[C@@H]([C@H]3COc4ccc(OC5CCCC5)cc4[C@H]3O)[C@@H](O)c2cc1OC	Cc1ccc(cc1)S(=O)(=O)NC[C@H]2C[C@@H]3CC[N@H]2C[C@@H]3CN4CCc5ccccc45
2D Structure	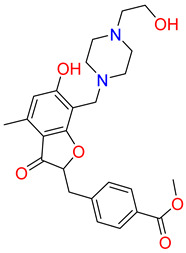	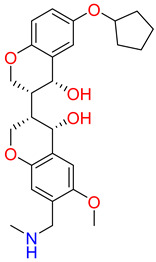	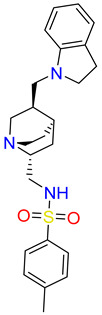

**Table 5:** In silico ADME property assessment of REF and identified SHP2 hits.

ADME Properties		Lig_1	Lig_6	Lig_14	REF (SHP099)	Cutoff
Absorption	WS (log mol/L)	−3.3	−4.28	−3.17	−3.84	<−10 insoluble to <0 highly soluble
	Caco-2 Permeability (log cm/s)	0.18	1.042	1.66	0.999	>0.90
	IA human (% abs)	63.17	90.35	96.62	92.23	>30
	SP (log KP)	−2.74	−2.75	−2.81	−3.01	>−2.5
	P-glycoprotein Substrate	Yes	Yes	Yes	No	No
	P-glycoprotein I inhibitor	No	Yes	Yes	No	No
	P-glycoprotein II inhibitor	No	No	Yes	No	No
Distribution	VDss (human)	0.856	0.857	1.152	0.865	<0.71 low to >2.81 high
	Fraction unbound (human)	0.262	0.138	0.146	0.308	Numeric (Fu)
	BBBP (logBB)	−1.153	−0.751	−0.262	−0.451	>0.3 high to <−1 poor
	CNS permeability	−3.685	−3.224	−2.606	−2.88	>−0.2 high to <−3 poor
Metabolism	CYP2D6 substrate	No	No	No	No	No
	CYP2D6 inhibitor	No	No	No	No	No
	CYP3A4 substrate	Yes	Yes	Yes	Yes	No
	CYP3A4 inhibitor	No	Yes	Yes	No	No
	CYP1A2 inhibitor	No	No	No	Yes	No
	CYP2C19 inhibitor	No	No	No	No	No
	CYP2C9 inhibitor	No	No	No	No	No
Excretion	TC (mL/min/kg)	1.312	1.145	1.005	0.66	Numeric (mL/min/kg)
	Renal OCT2 substrate	No	No	Yes	No	No

Abbreviations: WS—water solubility, IA—intestinal absorption, SP—skin permeability, VDss—volume of distribution at steady-state, BBBP—blood–brain barrier permeability, CNS—central nervous system, TC—total clearance, OCT2—Organic Cation Transporter 2.

**Table 6:** In silico toxicity profile assessment of REF and identified hits.

Toxicity Parameters		Lig_1	Lig_6	Lig_14	REF (SHP099)	Cutoff
Toxicity	AMES toxicity	No	No	No	No	Categorical (Yes/No)
	Max. tolerated dose (human)	−0.15	−0.12	−0.42	−0.18	>0.477 mg/kg/day
	hERG I inhibitor	No	No	No	No	Categorical (Yes/No)
	hERG II inhibitor	Yes	Yes	Yes	Yes	Categorical (Yes/No)
	Oral Rat Acute Toxicity (LD_50_)	2.944	2.72	2.61	2.932	Numeric (mol/kg)
	Oral Rat Chronic Toxicity (LOAEL)	2.852	1.76	1.86	0.508	Numeric (mg/kg_bw/day)
	Hepatotoxicity	Yes	No	Yes	Yes	Categorical (Yes/No)
	SS	No	No	No	No	Categorical (Yes/No)
	T. Pyriformis toxicity	0.29	0.294	0.33	0.513	>0.5 μg/L Toxic
	Minnow toxicity	1.488	1.498	1.107	2.056	<−0.3 Toxic

Abbreviations: hERG—human ether-à-go-go-related gene, LD_50_—Lethal Dose, LOAEL—lowest observed adverse effect, SS—skin sensitization.

## Data Availability

The original contributions presented in the study are included in the article/Supplementary Material, further inquiries can be directed to the corresponding authors.

## Supplementary Materials

The following supporting information can be downloaded at: https://www.mdpi.com/article/10.3390/ph17070935/s1, Figure S1. (**A**) Docking parameters validation using co-crystalized structure (grey) and selected docked pose (Blue) protein is shown in grey color. The RMSD value between both structures was 1.53 Å. (**B**) The enlarged view of the binding pattern of REF (SHP099); Figure S2. The binding pattern of identified Hits (Lig_1, Lig_6, and Lig_14) and selective allosteric site 1 inhibitor SHP099 (REF). (**A**) The superimposed view of Lig_1, Lig_6, Lig_14, and REF is shown in the left image. (**B**) The enlarged view is depicted in the right-side image. The protein is represented in grey, whereas hits are shown in yellow, pink, and green. All the hydrogen atoms were deleted except the polar hydrogen atoms for clear visualization. Table S1. Receptor-based pharmacophore models generated from PDB: 6CMR. Table S2. Parameters used for the generation of a drug-like database. Table S3. List of potential compounds obtained from molecular docking.
