# Supplementary material for: Discovery of Novel Allosteric SHP2 Inhibitor Using Pharmacophore-Based Virtual Screening, Molecular Docking, Molecular Dynamics Simulation, and Principal Component Analysis
Source: Pharmaceuticals (Basel). 2024 Jul 12;17(7):935. doi: 10.3390/ph17070935 (PMC11280062; doi:10.3390/ph17070935)
Supplement: Supplementary file 1 [file pharmaceuticals-17-00935-s001.zip › pharmaceuticals-3088090-supplementary.pdf]

Table S1. Receptor-based pharmacophore models generated from PDB: 6CMR

Pharmacophore Models Summary			
**Pharmacophore**	**Number of Features**	**Feature Set**	**Selectivity Score**
Pharmacophore 01	5	ADHHP	10.993
Pharmacophore 02	5	DDHHP	9.983
Pharmacophore 03	5	ADHHP	9.766
Pharmacophore 04	4	ADHP	8.468
Pharmacophore 05	4	ADHP	8.468
Pharmacophore 06	4	AHHP	8.455
Pharmacophore 07	4	AHHP	7.554
Pharmacophore 08	4	ADHH	7.467
Pharmacophore 09	4	ADHH	7.074
Pharmacophore 10	4	ADDH	7.074

Table S2. Parameters used for the generation of a drug-like database.

Lipinski's rule of five (ROF)		ADMET descriptors	
**Parameters**	**Cut off**	**Parameters**	**Cut off**
Number of hydrogen bond donors	≤5	Absorption level	0 (Good)
Number of hydrogen bond acceptors	≤10	Solubility level	3 (Good)
Molecular weight (Da)	≤500	Blood-brain barrier level	3 (Low)
AlogP value	≤5	CYP2D6 prediction	False
		Hepatotoxic prediction	False

Table S3. List of potential compounds obtained from molecular docking.

Compound	Goldscore	Chemscore	2D structure
REF(SHP099)	55.33	-28.35	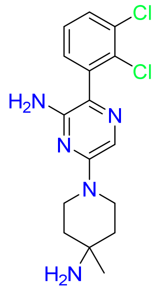
Lig_1	79.37	-37.47	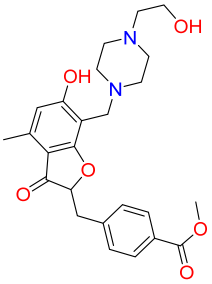
Lig_2	79.19	-23.49	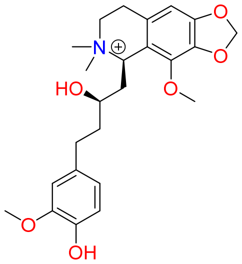
Lig_3	78.25	-22.15	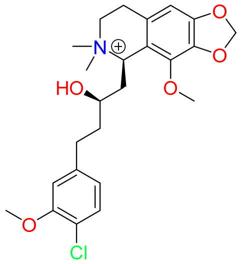
Lig_4	77.31	-35.70	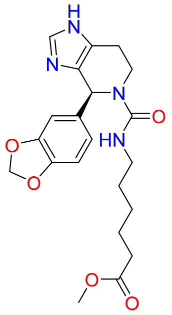

Lig_5	75.80	-39.59	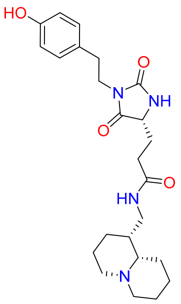
Lig_6	73.16	-20.03	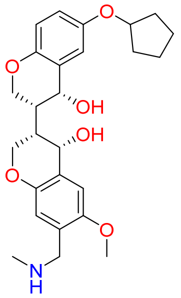
Lig_7	72.89	-45.07	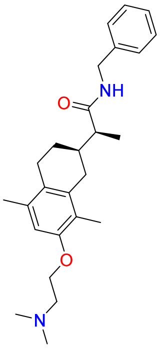
Lig_8	72.81	-44.14	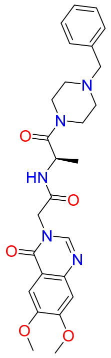
Lig_9	72.34	-57.78	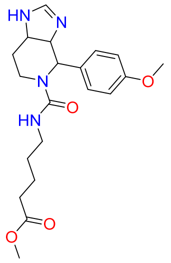

Lig_10	72.31	-51.88	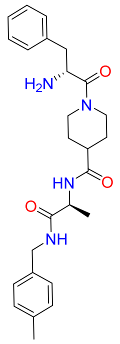
Lig_11	70.09	-27.68	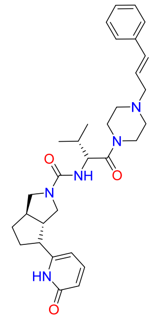
Lig_12	69.58	-37.20	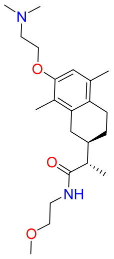
Lig_13	68.50	-28.30	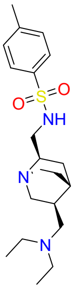
Lig_14	68.37	-26.43	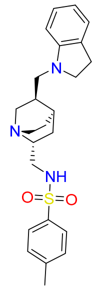
Lig_15	68.23	-53.61	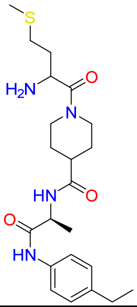

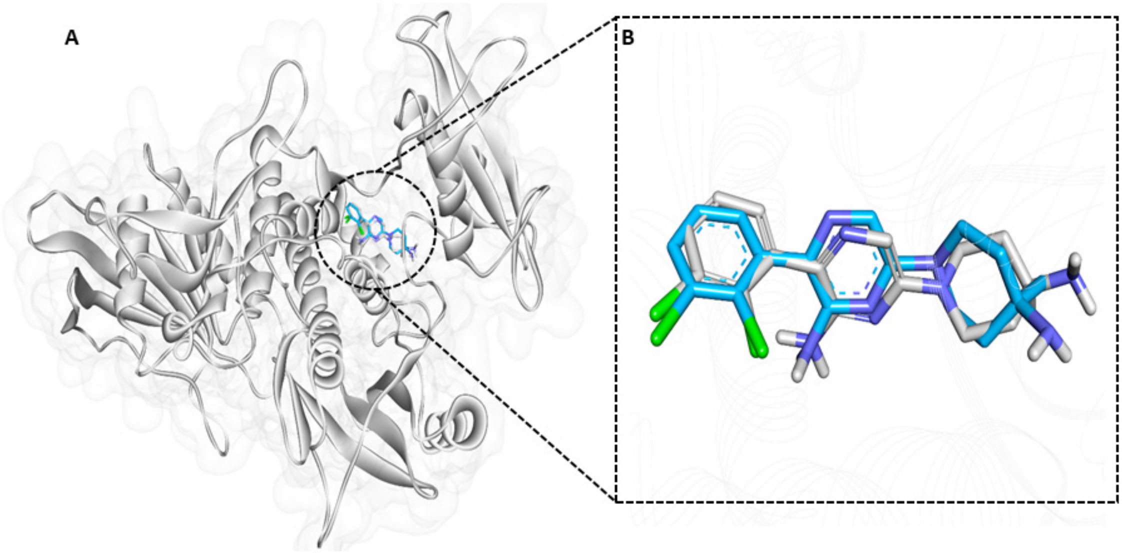

Figure S1. A) Docking parameters validation using co-crystalized structure (grey) and selected docked pose (Blue) protein is shown in grey color. The RMSD value between both structures was 1.53Å. B) The enlarged view of the binding pattern of REF (SHP099).

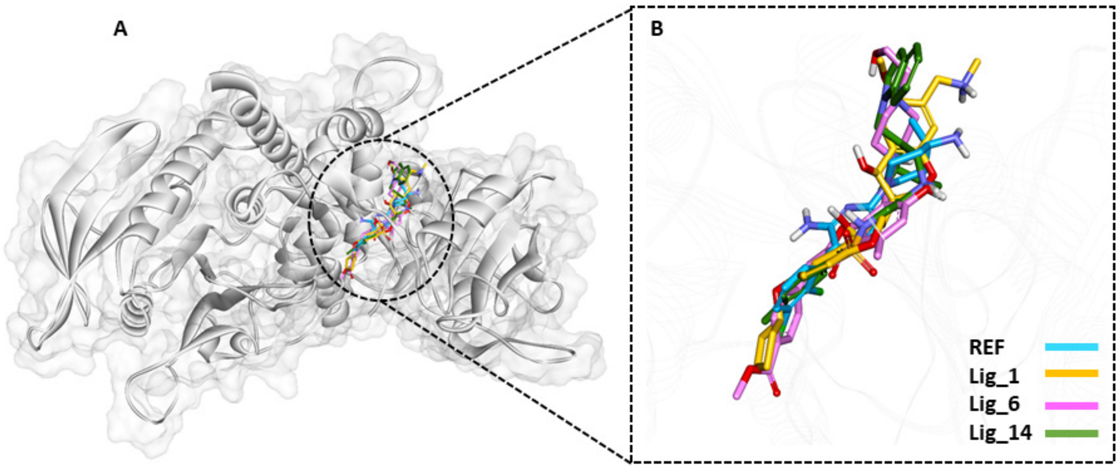

Figure S2. The binding pattern of identified Hits (Lig_1, Lig_6, and Lig_14) and selective allosteric site 1 inhibitor SHP099 (REF). (A) The superimposed view of Lig_1, Lig_6, Lig_14, and REF is shown in the left image. (B) The enlarged view is depicted in the right-side image. The protein is represented in grey, whereas hits are shown in yellow, pink, and green. All the hydrogen atoms were deleted except the polar hydrogen atoms for clear visualization.
